# Biomechanical evaluation of different medial column fixation patterns for valgus pilon fractures

**DOI:** 10.1186/s12891-024-07660-2

**Published:** 2024-07-11

**Authors:** Bing-Hao Wang, Bin-Bin Zhang, Zi-Ling Gong, Jiong Mei, Cong-Feng Luo, Yi Zhu

**Affiliations:** Department of Orthopaedic Surgery, Shanghai Sixth People’s Hospital, Affiliated to Shanghai Jiao Tong University School of Medicine, 600 Yishan Road, Shanghai, 200233 China

**Keywords:** Pilon fracture, Internal fixation, Biomechanical study, Fixation stability

## Abstract

**Background:**

The purpose of this study was to perform a biomechanical analysis to compare different medial column fixation patterns for valgus pilon fractures in a case-based model.

**Methods:**

Based on the fracture mapping, 48 valgus pilon fracture models were produced and assigned into four groups with different medial column fixation patterns: no fixation (NF), K-wires (KW), intramedullary screws (IS), and locking compression plate (LCP). Each group contained wedge-in and wedge-out subgroups. After fixing each specimen on the machine, gradually increased axial compressive loads were applied with a load speed of one millimeter per minute. The maximum peak force was set at 1500 N. Load-displacement curves were generated and the axial stiffness was calculated. Five different loads of 200 N, 400 N, 600 N, 800 N, 1000 N were selected for analysis. The specimen failure was defined as resultant loading displacement over 3 mm.

**Results:**

For the wedge-out models, Group-IS showed less displacement (*p* < 0.001), higher axial stiffness (*p* < 0.01), and higher load to failure (*p* < 0.001) than Group-NF. Group-KW showed comparable displacement under loads of 200 N, 400 N and 600 N with both Group-IS and Group-LCP. For the wedge-in models, no statistical differences in displacement, axial stiffness, or load to failure were observed among the four groups. Overall, wedge-out models exhibited less axial stiffness than wedge-in models (all *p* < 0.01).

**Conclusions:**

Functional reduction with stable fixation of the medial column is essential for the biomechanical stability of valgus pilon fractures and medial column fixation provides the enough biomechanical stability for this kind of fracture in the combination of anterolateral fixation. In detail, the K-wires can provide a provisional stability at an early stage. Intramedullary screws are strong enough to provide the medial column stability as a definitive fixation. In future, this technique can be recommended for medial column fixation as a complement for holistic stability in high-energy valgus pilon fractures.

## Introduction

Pilon fractures pose a challenge to orthopedic surgeons due to the high energy feature of both distal tibia fractures and concomitant soft tissue injuries. Surgeons commonly adopt a staged strategy in the treatment of pilon fractures, tailoring the specific treatment based on the fracture pattern and soft tissue involvement [1, 2]. In the treatment of pilon fractures caused by the valgus force injury mechanism [3, 4], the placement of an anterolateral plate through an anterolateral approach, minimizing irritation to the medial soft tissues, has been proved as an effective fixation strategy [5–7]. However, studies have reported a higher incidence of bone nonunion when utilizing a single anterolateral plate for fixation in pilon fractures [8–10].

Due to the potential problems of a single anterolateral plate in the fixation of pilon fractures, the significance of medial column stability was highlighted [10–12]. Non-fixation of the medial column would result in coronal plane malalignment and an increased risk of nonunion [10, 13]. Some studies even suggested that the medial column should be regularly fixed, thus a dual-plate strategy could maintain a normal alignment and reduce bone nonunion [10, 12, 14]. Given all the above-mentioned reasons, in the context of the staged treatment of high energy pilon fracture, it is necessary to choose an appropriate medial column implant in conjunction with an anterolateral plate. However, it is important to note that soft tissue injuries place limitations on the type and timing of medial column fixation in valgus pilon fractures [2, 15, 16]. Hence, the selection of medial column fixation pattern for such fractures should be considered as a holistic decision weighing medial column stability against soft tissue conditions.

While K-wires, intramedullary screws, and plates are viable options for the fixation of the medial column in pilon fractures, there is currently no consensus on the optimal choice of medial fixation [17, 18]. Besides, there is a void in biomechanical studies to evaluate the different medial fixation methods. Therefore, the objective of this study was to develop a case-based model for valgus pilon fractures and perform a biomechanical analysis to compare the effects of three commonly adopted medial fixation methods.

## Methods

### Mapping analysis

At a level-I trauma center, we conducted a search in the medical record system for patients diagnosed between January 2016 and December 2019 with the keywords “distal tibia fracture”, “lower tibia fracture” and “pilon fracture”. The following criteria were used for exclusion: (a) patients who were not treated by our department; (b) other fractures that were not pilon fractures; (c) concomitant non-avulsion fractures in other parts of the lower limb; (d) pathological fractures; (e) skeletal immaturity (age < 18 years); (f) patients without preoperative CT data; (g) fractures which did not meet the definition of valgus pilon fractures. The valgus pilon fracture is defined as a pilon fracture with residual valgus deformity of the tibia. Finally, 45 patients were considered eligible for this study.

The 3D-fracture mapping technique reported in previous literature was applied to the valgus pilon fractures [19]. The template was created using the CT imaging data of the left distal tibia of a 25-year-old healthy man. Then, the curves, which were combined into the fracture lines, were plotted on the surface of the template to mark the outline of each fragment. Finally, all fracture lines were summarized and overlapped to form a 3-dimensional fracture map. The heat map was generated by E-3D software (Central South University, Changsha, China) based on the spatial frequency of fracture lines, with blue to red indicating relatively low to high incidence.

### Modelling

**Fig. 1:**
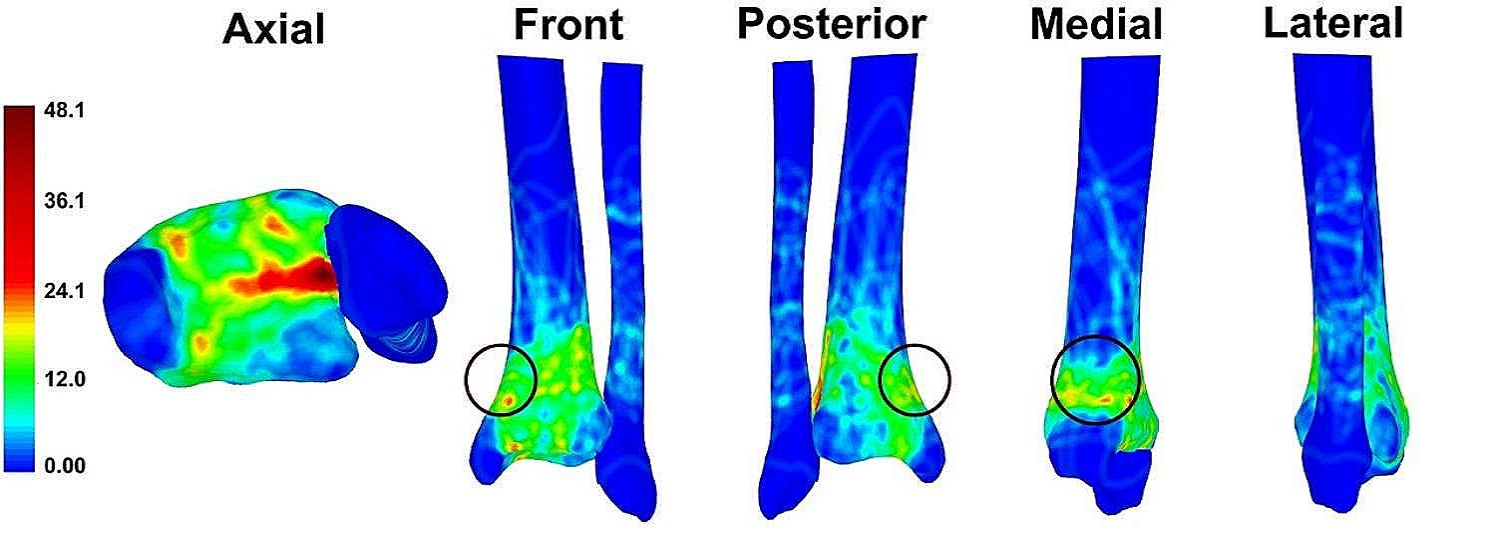
3D heat mapping superimposed with all valgus pilon fracture lines (*n* = 45). The common comminuted area of the medial column is marked with the black circle

**Fig. 2:**
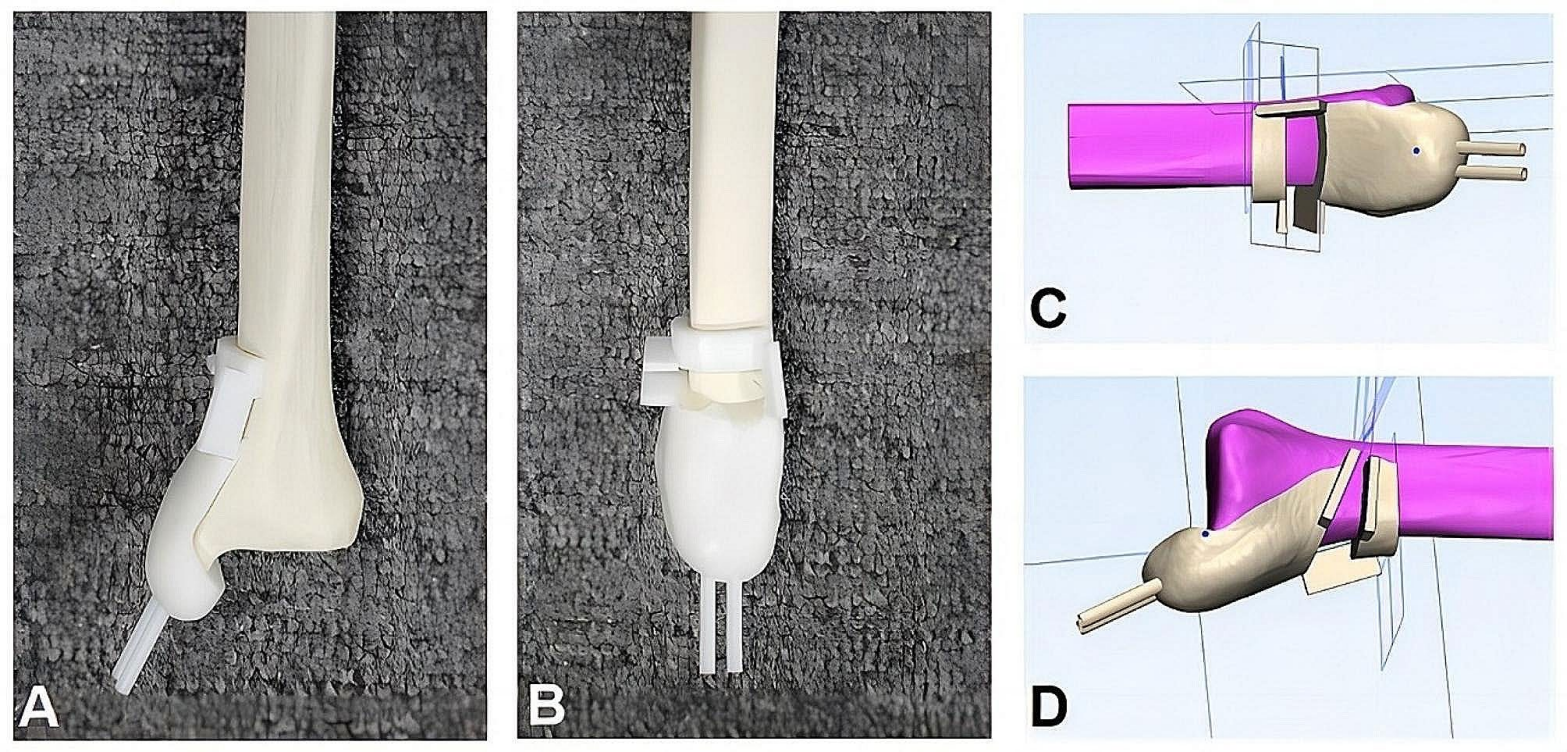
The polyamide cutting guide produced by the 3D printing technique

According to the fracture mapping delineations, a common comminuted area was identified in the medial column. The main fracture line clusters were from the inferior medial side to the superior lateral side of the distal tibia (Fig. 1). A medial wedge was then designed to mimic the medial comminution to show the varus deformity tendency. In order to produce the homogeneous fracture models, a polyamide cutting guide was produced by the 3D printing technique. The cutting guide was used to navigate a medial wedge-like osteotomy, as well as two sleeves which were designed for the medial parallel fixations, either K-wires or intramedullary screws (Fig. 2).

### Group

**Fig. 3:**
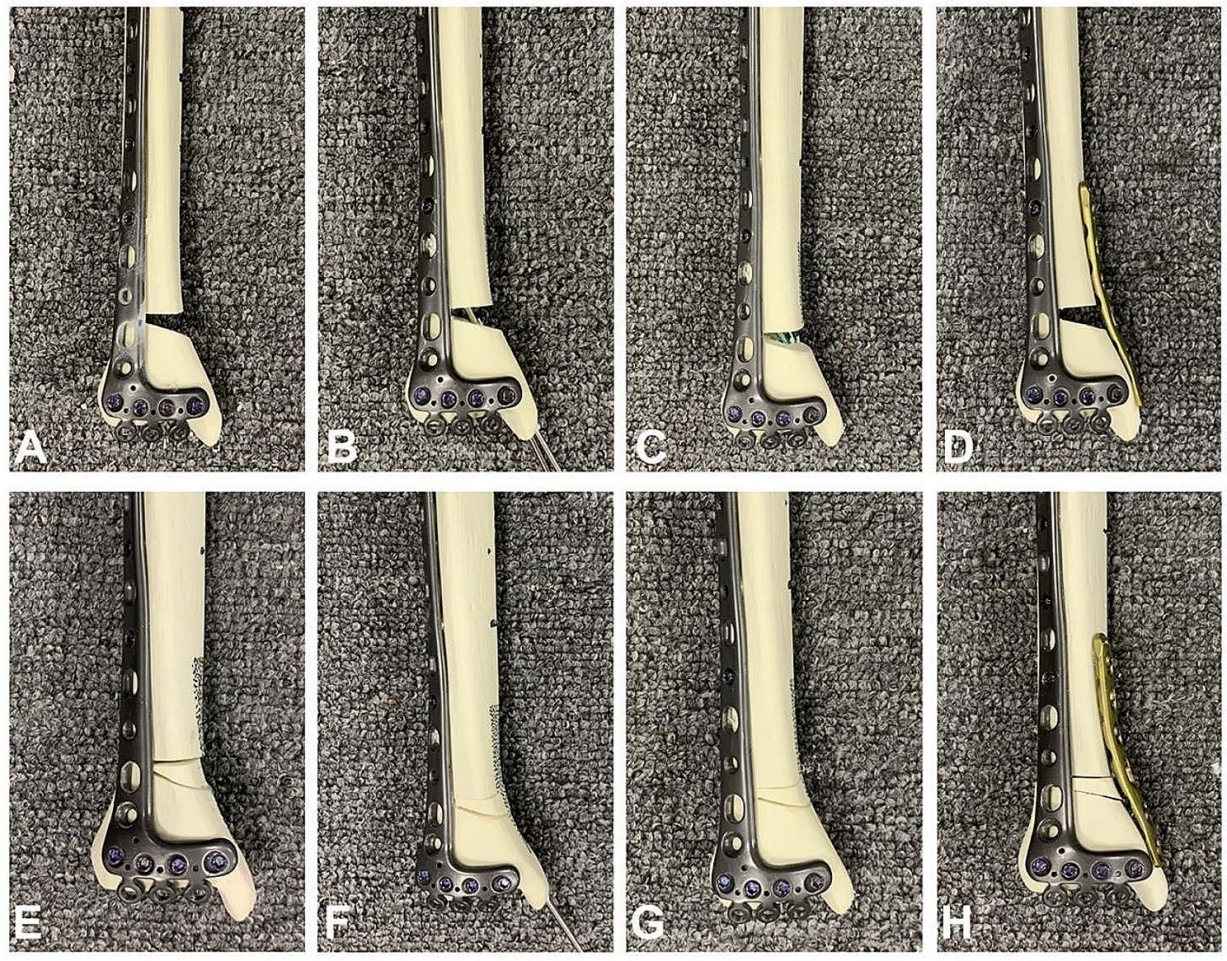
Illustration of the medial internal fixation patterns. Wedge-out models: (**A**) no fixation as a blank control (NF), (**B**) K-wires (KW), (**C**) intramedullary screws (IS), (**D**) locking compression plate (LCP); Wedge-in models: (**E**) no fixation as a blank control (NF), (**F**) K-wires (KW), (**G**) intramedullary screws (IS), (**H**) locking compression plate (LCP)

In this study, 48 right, synthetic, adult-sized tibiae models (Synbone, type 1110. Synbone AG, Malans, Switzerland) were used to produce valgus pilon fracture models. The models were manufactured by performing accurate osteotomy with a 3D-printed cutting guide. Subsequently, each model was randomly assigned to one of four groups: no fixation as a blank control (NF), K-wires (KW), intramedullary screws (IS), and locking compression plate (LCP). According to whether the model had a wedge-shaped bone block, each group was further divided into bony wedge-in (with a wedge-shaped bone block) and wedge-out (without a wedge-shaped bone block) subgroups, with 6 specimens in each subgroup (Fig. 3).

In Group KW, two 2.5 mm K-wires, which were in the center of the medial malleolus medullary cavity, were inserted from the medial side all the way to penetrate the far cortex of the tibial shaft. In Group IS, two long 3.5 mm parallel intramedullary screws were inserted into the holes predrilled by a 2.8 mm drill bit. In Group LCP, a 3.5 mm metaphyseal locking compression plate (Synthes GMBH, Oberderf, Switzerland), lying on the anteromedial surface of the distal tibia, was fixed with 3.5 mm screws for buttress function [18]. An anterolateral plate (Biomet Trauma, Indiana, USA) for distal tibial was utilized routinely to bridge the metaphyseal and diaphyseal parts of the tibia.

### Test

**Fig. 4:**
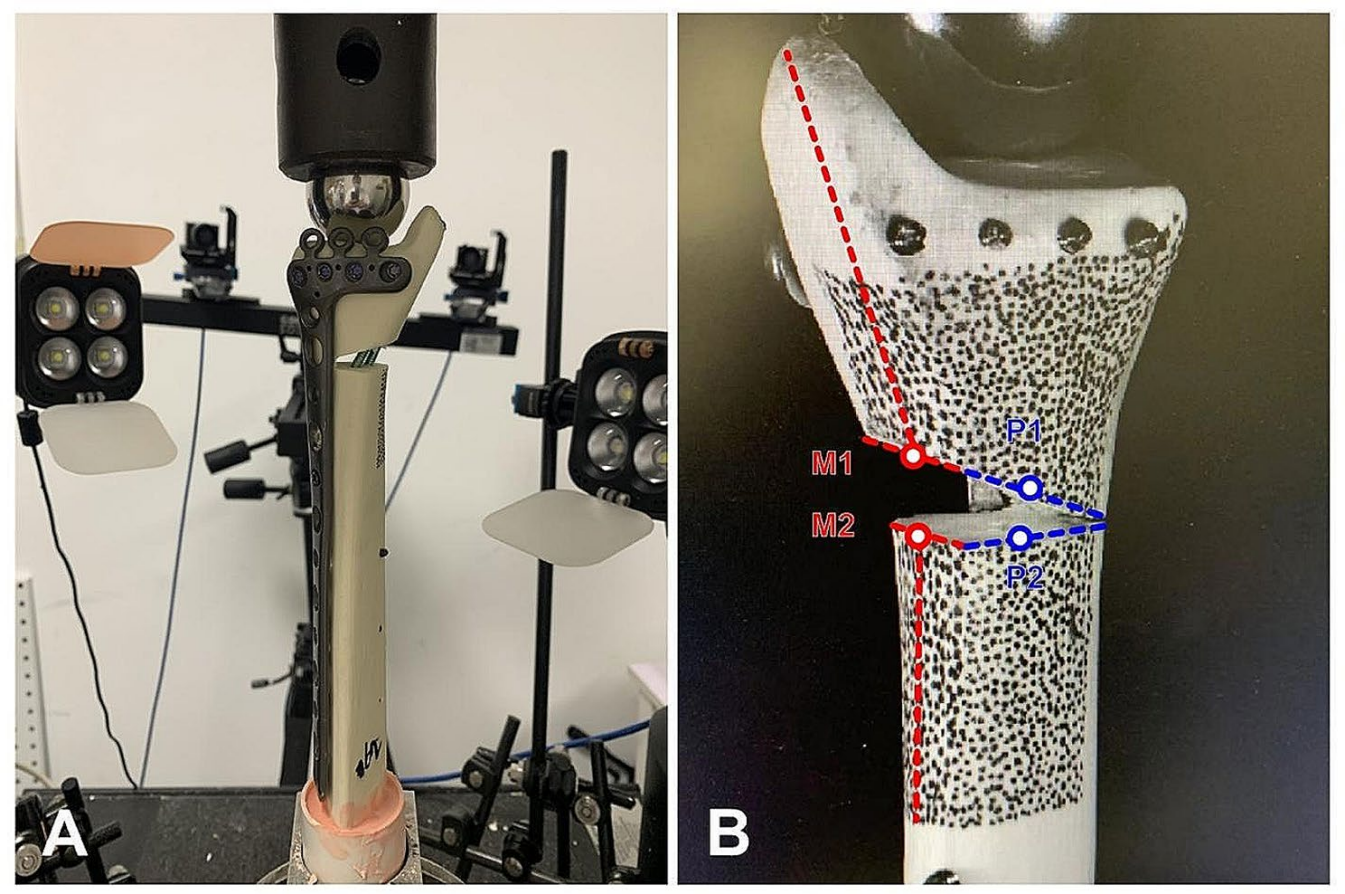
Schematic diagram of biomechanical test. (**A**) The load was applied to the distal end of the tibia through a metal ball. (**B**) Measurement of medial and posterior displacement

Each tibia model was cut with a power saw at the mid-shaft part of the tibia for biomechanical tests. Then, the tibia model was potted with die stone (Heraeus Kulzer Dental Ltd, China) vertically in an upside-down pattern in a material-testing machine (Instron 5569, Instron, Norwood, MA, USA) for testing. The load was applied to the distal end of the tibia through a metal ball (stainless steel, with a diameter of 3 cm) (Fig. 4). Both the medial and posterior surfaces of the tibia metaphyseal were manually painted in form of black random speckle patterns as a permanent marker. A VIC-3D system (XR-9 M; Correlated Solutions Company, WF, USA), which is based on the theory of digital image correlation was used to record relative displacement among speckles [20]. The medial displacement between point M1 and its opposite point M2 at about the midpoint of the medial wedge surface was measured as well as the posterior displacement between point P1 and its opposite point P2 at about the midpoint of the posterior wedge surface (Fig. 4). The models were axially loaded by an Instron test system (Instron, Norwood, MA, USA). After fixing each specimen on the machine, gradually increased axial compressive loads were applied to each specimen with a load speed of one millimeter per minute. The maximum peak force was set at 1500 N because the pretest had found that no failure of bone-implant construction, which was defined as plate loosening or deformation, and screw loosening or breakage, would occur within this limit. Load-displacement curves were generated for each via Bluehill 2 software (2.17.649, Instron, Norwood, MA, USA), and the axial stiffness was calculated from the linear portion of this load–displacement curve. Five different loads of 200 N, 400 N, 600 N, 800 N, 1000 N were selected for analysis. The specimen failure was defined as resultant loading displacement over 3 mm. The real-time loading value of specimen failure was recorded.

### Statistical analysis

The distribution of continuous data was compared using Mann-Whitney U test or Kruskal-Wallis test respectively. All statistics were computed with the use of SPSS (26.0; IBM Corp., Armonk, NY, USA). Statistical significance was set at *p* < 0.05. Bonferroni adjustments were performed for multiple comparisons.

## Results

### Displacement under different loads

**Fig. 5:**
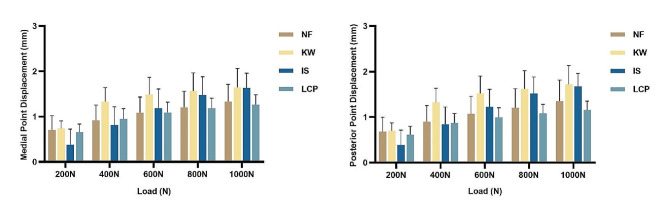
The displacement in wedge-in models

**Fig. 6:**
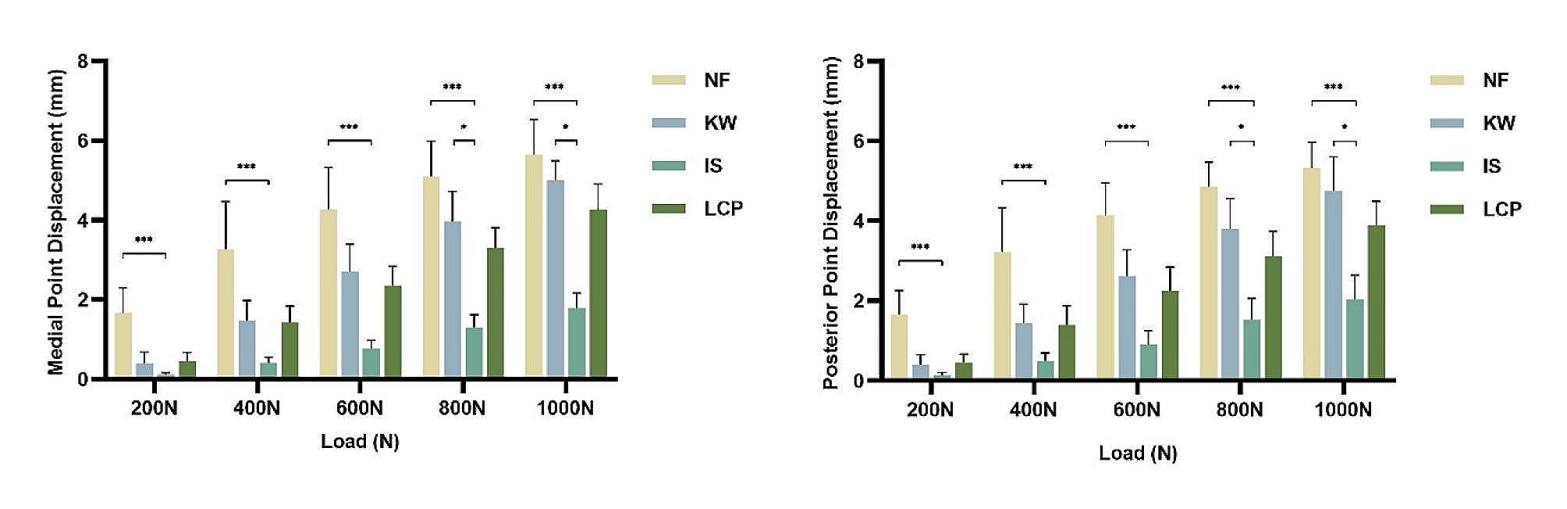
The displacement in wedge-out models

Both the medial and posterior sides of the wedge showed similar displacement results, indicating that these sides of the fracture wedge experienced a similar stress environment under load (Figs. 5 and 6).

For the wedge-in models, there was no statistical difference in displacement among the different groups (Fig. 5), indicating the anatomical reduction of the medial column could provide enough biomechanical stability for the whole device.

For the wedge-out models, Group-NF showed the most displacement as expected, while Group-IS showed the least. The displacement of Group-IS was statistically less than Group-NF (all *p* < 0.001). Under loads of 800 N and 1000 N, the Group-KW showed more displacement than Group-IS (*p* < 0.05). The Group-KW showed comparable displacement under loads of 200 N, 400 N and 600 N with other two fixation patterns, which were Group-IS and Group-LCP. The displacement of Group-LCP appeared to be more than that of Group-IS, but the difference was not statistically significant (Fig. 6).

### Axial stiffness

**Fig. 7:**
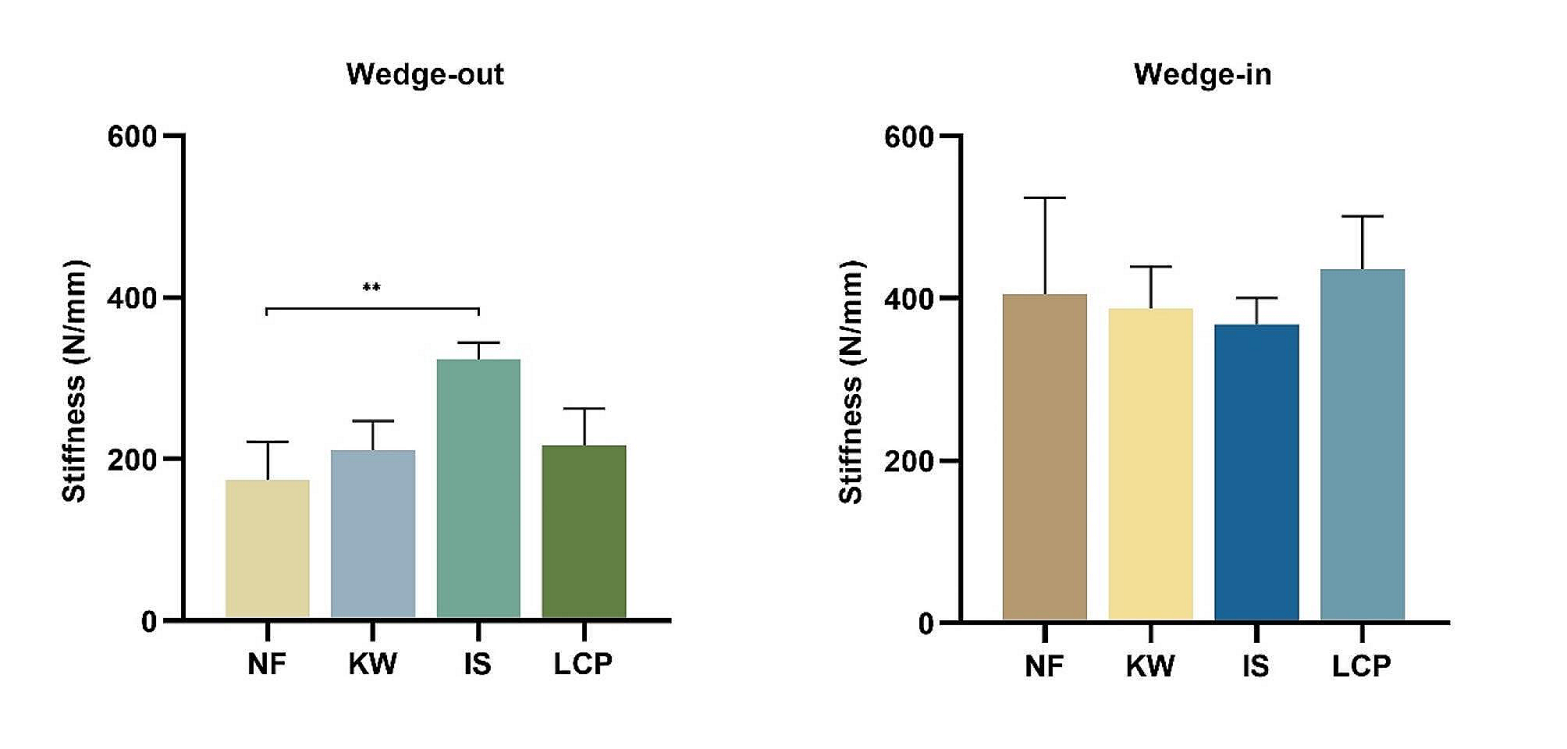
The stiffness of different fixation patterns

For the wedge-in models, there was no significant difference in the axial stiffness among the four groups. For the wedge-out models, Group-IS had the highest axial stiffness, which was statistically higher than that of Group-NF (*p* < 0.01). There was no significant difference in the axial stiffness among the other three groups. (Fig. 7).

### Load to failure

**Fig. 8:**
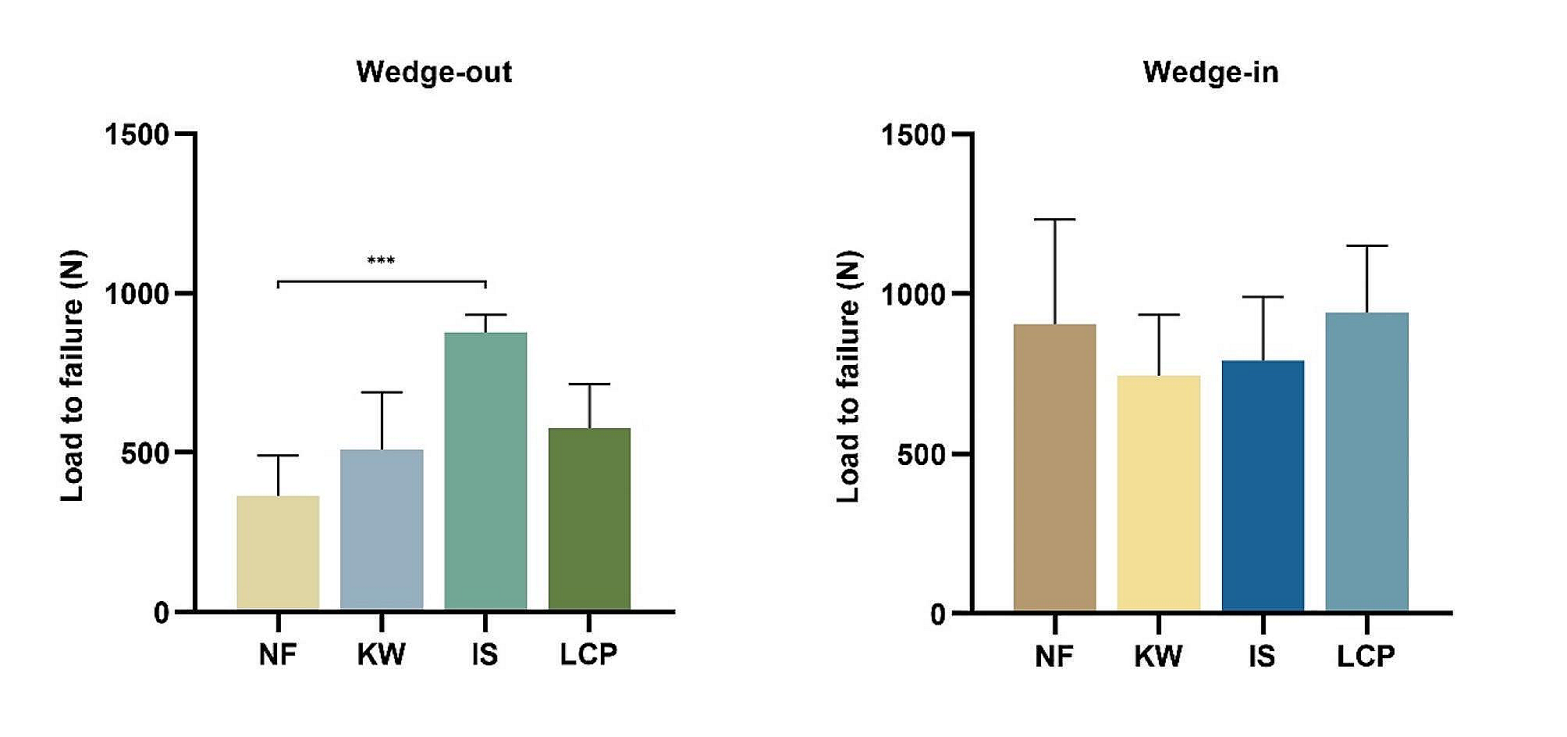
The load to failure of different fixation patterns

The loads to failure (displacement over 3 mm) were shown as Fig. 8. For the wedge-in models, there was no significant difference among the four groups. For the wedge-out models, Group IS had the highest load to failure and Group NF had the lowest load to failure, the difference between the two groups was statistically significant (*p* < 0.001).

## Discussion

Staged treatment, along with meticulous soft tissue management, is currently considered as the standard strategy for high energy pilon fractures [1, 2, 21]. From a biomechanical perspective, it seems better to place an anterolateral plate for pilon fractures with valgus injury force mechanism [22]. However, the effectiveness of using a single anterolateral plate for valgus pilon fractures is still controversial. A higher incidence of nonunion was observed in pilon fractures treated with anterolateral plates compared to medial plates [8–10]. Furthermore, increased callus formation was observed in pilon fractures treated with a single anterolateral plate, which might indicate mechanical instability according to Perren’s theory [13]. Consequently, some scholars have proposed the concurrent application of medial column fixation, in order to increase stability and avoid bone nonunion [10, 12, 14].

Based on 3D-mapping images of pilon fractures, we identified a common comminuted area in the medial column. In our study, a wedge was designed to mimic the state of comminution or non-anatomic reduction of the medial column. Based on our biomechanical study, the fracture displacement was largest when the wedge was removed in the scenario of a single anterolateral plate. However, the biomechanical stability of the entire internal fixation system increased when the medial column was fixed in the form of fragment wedge-in condition, regardless of the fixation methods. This was consistent with existing clinical findings that the absence of medial column fixation is associated with a higher likelihood of fixation instability, resulting in bone nonunion [10]. Therefore, from the biomechanical perspective, in order to pursue a stable biomechanical capacity, a medial column fixation should be performed in addition to an anterolateral plate for valgus pilon fractures.

Valgus pilon fractures often present in combination with severe soft tissue damage of the medial side of distal tibia, which increases the risk of postoperative complications [5]. Therefore, appropriate medial column internal fixation should be placed to not only avoid soft tissue irritation as much as possible, but also provide enough biomechanical stability [14, 15, 23]. In our wedge-out model, the K-wires provided comparable stability with plates and intramedullary screws under lower loads, indicating that the application of the K-wires was effective at the early stage if the medial soft tissue was not available for further intervention. Under higher loads, K-wires were not as strong as intramedullary screws, which provided the best biomechanical properties. Thus, in order to create a better and stronger biomechanical environment, replacing K-wires with other medial column fixations might be recommended as the medial soft tissue is appropriate for further intervention.

Intramedullary screws have been reported as viable implants for medial column fixation with satisfying early clinical outcomes [17]. In the wedge-out group of our study, intramedullary screws even showed better stability than plates, which could be explained from three aspects. Firstly, in our study, long solid screws were selected for intramedullary fixation, which had great pull-out force and ultimate bending moment [24]. Secondly, the intramedullary screw is biomechanically stronger than the eccentric location of the plate in the bone, based on the previous comparison of nail and plate. Finally, long intramedullary screws can act a role like a kick-stand mechanism, which might be another reason to be biomechanically superior in fixation than the LCP. Anyway, intramedullary screws meet the biomechanical demands of medial column fixation and reduce local vascularity damage compared to plates. In summary, intramedullary screws provide a minimally invasive strategy with better biomechanical capacity for medial column fixation in valgus pilon fractures.

This study has some limitations. Firstly, rather than cadaveric bone, synbone was used with the advantage of the homogeneity of the mechanical properties and bone geometry, reducing the variability between specimens. However, synbone inadequately simulated the mechanical construction of bone with trabecular system in the internal fixation system. Secondly, the model was loaded with a continuously increasing force directly to failure to test the strength of the fixation construct. Considering that patients with pilon fractures could not walk for long periods of time immediately after fixation, we did not test the fixation construct for cyclic loading to failure.

## Conclusions

Functional reduction with stable fixation of the medial column is essential for the biomechanical stability of valgus pilon fractures and medial column fixation provides the enough biomechanical stability for this kind of fracture in the combination of anterolateral fixation. In detail, the K-wires can provide a provisional stability at an early stage. Intramedullary screws are strong enough to provide the medial column stability as a definitive fixation. In future, this technique can be recommended for medial column fixation as a complement for holistic stability in high-energy valgus pilon fractures.

## Data Availability

The datasets used or analyzed during the current study are available from the corresponding author on reasonable request.
